# Minimally invasive surgical resection reduces one-year mortality, especially in high-risk colon cancer patients: an emulated trial

**DOI:** 10.1016/j.eclinm.2025.103683

**Published:** 2026-02-02

**Authors:** Daniel O'Leary, Camille Maringe, Sara Benitez-Majano, Clemence Leyrat, Bernard Rachet, Manuela Quaresma

**Affiliations:** a Colorectal Unit, Surgical Department, Queen Alexandra Hospital, Cosham, Portsmouth, UK; b Inequalities in Cancer Outcome Network (ICON), Department of Health Services Research and Policy, Faculty of Public Health and Policy, London School of Hygiene & Tropical Medicine, 15-17 Tavistock Place, WC1H 9SH, London, UK; c Department of Medical Statistics, Faculty of Epidemiology and Population Health, London School of Hygiene & Tropical Medicine, Keppel Street, WC1E 7HT, London, UK

**Keywords:** Minimally invasive surgical resection, Open surgical resection, Colon cancer, Inequalities

## Abstract

**Background:**

Comorbidity, older age, frailty and socioeconomic deprivation are associated with higher risk of complications and death following resection for colon cancer. Minimally invasive surgical (MIS) resection is associated with earlier recovery, fewer postoperative complications and potentially lower early mortality than open surgical (OS) resection. We investigated the likelihood of receiving MIS vs OS resection by patient characteristics, and whether MIS resection reduces mortality after elective resection for colon cancer.

**Methods:**

We analysed cancer registration data linked to secondary care records of 21,931 patients diagnosed with stage I-III colon cancer in NHS Trusts in England, in 2021 and 2022. We focused on elective operations completed as either MIS resections or OS resections. We used an emulated trial to estimate the impact of MIS resection on one-year mortality (expressed as Average Treatment Effect) compared with OS resection. Inverse-probability-weights with regression adjustment ensured comparability between the surgical groups.

**Findings:**

MIS resection was attempted in 18,264 (83.3% of 21,931) patients and completed in 16,271 (74.2% of 21,931), among whom higher levels of deprivation, frailty, comorbidity and stage at diagnosis were independently associated with lower odds of receiving MIS resection. Observed one-year mortality was 7.7% after OS resection (436 deaths among 5660) vs 2.9% after MIS resection (472 deaths among 16,271). In the emulated trial, the average treatment effect of MIS resection was a reduction in one-year mortality from 6.8% to 3.0%; with the largest absolute reductions among patients aged 85 years or more, frail patients, and those with major comorbidities.

**Interpretation:**

The emulated trial confirms that MIS resection for colon cancer reduces mortality at one year, compared with OS resection. However, patients in higher-risk groups, who were most likely to benefit from MIS resection, were less likely to receive it. The NHS needs to eliminate ongoing inequalities in optimal surgery for colon cancer.

**Funding:**

10.13039/501100000289Cancer Research UK C7923/A29018; 10.13039/501100000265Medical Research Council MR/T032448/1 and MR/W021021.

**Research in context**

**Evidence before this study**

Survival from colon cancer is worse in England than in many European countries. Minimally Invasive Surgical (MIS) resection improves early surgical outcomes for colon cancer and is increasingly replacing Open Surgical (OS) resection in England. Population studies suggest that MIS resection is associated with reduced mortality. We searched PubMed for studies examining inequalities in the use of MIS resections in England from database inception to June 30, 2025, without English language restriction, using the search terms (“minimally invasive” OR “laparoscop∗”) AND “inequal∗” AND “England”, without language or publication date restrictions. We found two articles reporting persistent regional and sociodemographic variations in access to MIS in England.

**Added value of this study**

This is the first study to use an emulated trial design based on national population data to determine whether choice of surgical technique (MIS resection vs OS resection) has an impact on mortality after resection for Stage I-III colon cancer patients, overall and for ‘high-risk’ groups. It is unlikely that a randomised controlled trial allowing analysis of effects in patients from ‘high-risk’ groups would be undertaken. The emulated trial, demonstrates that, compared with OS resection, MIS resection reduces mortality one-year post resection especially among patients who are frail, comorbid, older or more deprived. However, in 2021–2022, these patient groups were least likely to have received it.

**Implications of all the available evidence**

This study supports the switch from OS resection to MIS resection for colon cancer including among higher-risk patients. More generally, inequalities in selection for cancer treatment may contribute to worse outcomes for cancers in England compared with other countries. Selective use of innovative therapies may introduce new inequalities in cancer care.

## Introduction

Patients with colorectal cancer in England have poorer survival than those in other countries with similar income and health systems.^1^^,^^2^ Between 2010 and 2012, rates of surgical resection in England were lower than in Denmark, Norway and Sweden.^3^ Three-year stage-specific survival paralleled the probability of resection. A further study highlighted higher rates of surgical resection in Denmark than in Yorkshire, UK.^4^ In two of these studies, disparities in resection rates widened with age.^3^^,^^4^ Poorer survival in England may reflect in part, shortfalls in treatment or inequalities in delivery of resectional surgery.

Randomised Controlled Trials (RCTs) have shown that laparoscopic minimally invasive surgical (MIS) resection for colorectal cancer delivers comparable long-term survival to open surgical (OS) resection.^5^, ^6^, ^7^ However, laparoscopic MIS resection is associated with smaller incisions, faster recovery and shorter hospital stay.^5^, ^6^, ^7^, ^8^ Population-based studies have reported lower mortality after MIS than OS resection for colon cancer.^9^, ^10^, ^11^ Increasing surgical experience, improvements in MIS technology and selection-for-treatment bias might account for this finding, which was not observed in RCTs.^5^, ^6^, ^7^, ^8^

Postoperative mortality, after major resection for colorectal cancer, is higher among patients who are older, comorbid or frail, as well as those from more socio-economically deprived areas.^12^, ^13^, ^14^ Retrospective studies and systematic reviews suggest that MIS resection may be especially beneficial in high-risk groups including patients who are older,^15^ obese or comorbid.^16^ In England, laparoscopic MIS resection has been supported by the National Institute for Health and Care Excellence (NICE) since 2006, and was advanced by a national training scheme for existing consultant colorectal surgeons in 2009–2012.^17^, ^18^, ^19^ Between 2013 and 2022, MIS resection increased from 48% to 74% of colorectal cancer resections reported to the National Bowel Cancer Audit (NBOCA).^20^^,^^21^ However, population-based observational studies in England demonstrate that comorbid patients and those from more deprived socioeconomic backgrounds were less likely to receive laparoscopic resection for colorectal cancer.^22^^,^^23^ Focusing on colon cancer, reports from Switzerland and the USA have highlighted inequalities in implementation of MIS colon cancer resection among the old, comorbid, socioeconomically deprived and those living in rural vs urban settings.^24^^,^^25^

In this study, we investigated implementation and one-year mortality after MIS vs OS resection for colon cancer among higher- and lower-risk patient groups in England. We emulated a target trial using the 2021–2022 national colon cancer dataset in England to determine whether elective MIS resection for colon cancer reduces one-year mortality compared with OS resection.

## Methods

### Data sources

Information on all adult (aged 15–99 years) patients with colon cancer (International Classification of Diseases for Oncology, third edition, ICD-O-3 codes 18.0, 18.2–18.9) diagnosed in England between 1 January 2021 and 31 December 2022 was extracted from the National Cancer Registration and Analysis Service (NCRAS) records linked to Hospital Episode Statistics (HES), including information on patient and tumour characteristics, cancer stage, diagnostic routes, treating NHS Trust and procedures undergone (coded using the procedural classification OPCS Classification of Interventions and Procedures, version 4, used by the National Health Service, NHS England). Ascertainment of vital status was complete up to the 31st December 2023.

### Aim

The aim of this emulated trial was to estimate the potential causal effect of MIS resection vs OS resection on one-year mortality from any cause.^26^

### Design of the emulated trial

**Fig. 1:**
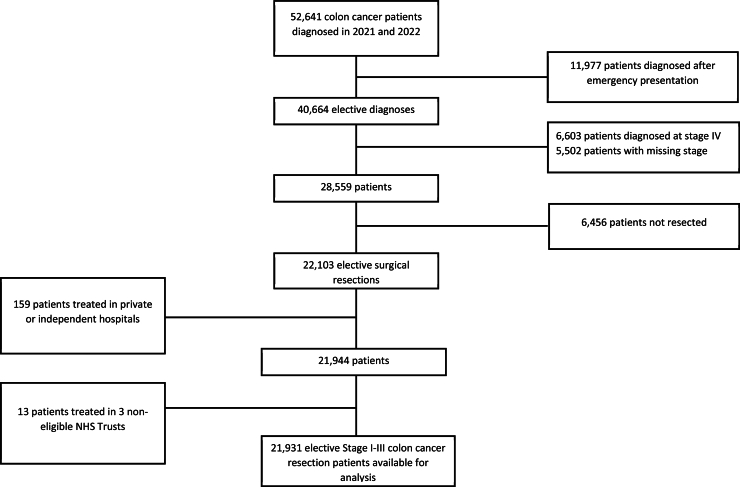
**Flow chart of colon cancer patients included in the analysis, England, 2021–2022**.

Among 52,641 patients with colon cancer (Fig. 1), we emulated a hypothetical target trial in which each patient would be randomised to MIS resection or OS resection. Trial emulation involved describing protocols for (i) the ‘target’ RCT if feasible and (ii) an emulated trial that was as close to this as possible within the limitations of the NCRAS data. We developed a strict protocol to reproduce as closely as possible the conditions of an RCT, following the framework developed by Hernán and Robins^26^ and supported by the web-based tool CERBOT^27^ (Supplementary Table S1).

### Eligibility and exclusion criteria

We excluded 11,977 patients diagnosed through an emergency route, as defined by the algorithm presented in Elliss Brookes et al.,^28^ 6,603 patients diagnosed with metastatic disease (Stage IV), 5,502 patients with missing stage, and 6,456 patients not resected. Among those undergoing resection, we included only those of morphological types “adenocarcinoma” or “carcinoma”. Patients with resections for cancers of the appendix were not included (ICD-O-3 code 18.1). Of the 22,103 elective colon cancer patients undergoing surgical resection, we excluded 159 patients who were treated in private or “independent” hospitals or for whom clear linkage to an NHS Trust could not be reliably identified, three patients who were treated in one NHS Trust that did not have a record of carrying out both MIS and OS resections for colon cancer, and 10 patients who underwent colon cancer surgery in two NHS Trusts that recorded ten or fewer colon cancer resections a year during the study period. Based on these inclusion/exclusion criteria, data on 21,931 patients treated at 124 NHS Trusts was used in descriptive analyses and the emulated trial (Fig. 1).

### Treatments and assignment

We contrast two treatments: operations completed as MIS resection vs OS resection for colon cancer. Resectional surgery (resection) was defined as segmental surgical removal of primary tumour, independent of surgical intent or oncological outcome (curative or palliative), performed in an NHS Trust within six months of the cancer diagnosis.^3^ Procedures were flagged as MIS when there was a laparoscopy OPCS code (Y75.1 “Laparoscopically assisted approach to abdominal cavity”, or Y75.2 “Laparoscopic approach to abdominal cavity not elsewhere classified”, or Y75.3 “Robotic minimal access approach to abdominal cavity”, or Y75.4 “Hand assisted minimal access approach to abdominal cavity”, or Y75.5 “Laparoscopic ultrasonic approach to abdominal cavity”, or Y75.8 “Other specified minimal access to abdominal cavity”, or Y75.9 “Unspecified minimal access to abdominal cavity”, or Y76.5 “Robotic assisted minimal access approach to other body cavity”, or Y76.8 “Other specified minimal access to other body cavity” or Y76.9 “Unspecified minimal access to other body cavity”) recorded for that patient on the day of the colon cancer surgery (resection) at the same NHS Trust. Robotic resections accounted for 10% of all MIS resections. Procedures were categorised as OS resections when there was no flag for MIS approach for the same individual on the date of resection or where the code for conversion (Y71.4 “Failed minimal access approach converted to open”) was used.

### Adjustment variables

Age was categorised into five groups: 54 years and younger, 55–64, 65–74, 75–84, and 85 years and older.

Socioeconomic status of patients at the time of colon cancer diagnosis was represented by the Income Domain quintiles from the Index of Multiple Deprivation (IMD 2015) score of the Lower Super Output Area of residence (1-least deprived, up to 5-most deprived).^29^

Cancer stage at diagnosis (I-IV) was used as defined in the NCRAS ‘Stage_best’ variable.^30^

Major comorbidity was deemed present when patients had a hospital record of any of the following in the six years preceding cancer diagnosis: myocardial infarction, heart failure, cerebro-vascular disease, chronic obstructive pulmonary disease, diabetes with complications, chronic renal disease, severe liver disease, or obesity. Relevant comorbidity codes were identified using a previously described algorithm.^31^

An indicator of frailty was derived from the linked HES data using the Hospital Frailty Score Index. The index was developed and validated by Gilbert and colleagues to identify older patients admitted to hospital with different levels of frailty risk (low: score 0–4, moderate: score 5–14 and high: score 15 or more) based on their service use history and ICD-10 codes in the two years prior to their cancer diagnosis.^32^

### Statistics

1. Descriptive analysis and association of MIS vs OS resection with patient/tumour characteristics

Descriptive statistics were calculated using absolute and relative frequencies (n, %) for each variable. Pearson's Chi-square tests were used to examine the association between surgical approach (MIS vs OS) and patient or tumour characteristics (age, sex, deprivation quintile, TNM stage, major comorbidity status and frailty risk category). We then used a mixed-effects logistic regression model to estimate the association between the type of resection performed (MIS or OS) and each of these characteristics, mutually adjusting for the potential confounding effects of all included covariates. Clustered standard errors were used to account for intragroup correlation among patients treated within the same NHS Trust, recognising that observations within Trusts are not independent. Results are presented as odds ratios, adjusted for all covariates, and are reported for the whole cohort as well as stratified by stage at diagnosis.

2. Average Treatment Effects of MIS vs OS resection on one-year mortality

The causal treatment effect to be estimated (*estimand*) was the risk difference in one-year mortality between MIS resection vs OS resection. We used the potential outcomes framework where each individual is considered to have two outcomes: one for each surgical treatment—one of which corresponds to the observed surgical treatment (observed outcome), and the other is the hypothetical outcome (or counterfactual). To account for confounding in the assignment of treatment (MIS vs OS), we utilised the inverse-probability-weighted regression adjustment (IPW-RA) approach. This method involved three main steps:

I) We first built a mixed-effects logistic regression model, with clustered standard errors on NHS Trust, to predict the probability that each patient receives either MIS or OS resection based on their individual characteristics and the specific features of their tumours (age, sex, deprivation quintile, TNM stage, major comorbidity status and frailty risk category).
II) Next, we assigned a weight to each patient based on the inverse of their probability of receiving MIS resection. The assignment of these weights reinforces the groups of patients who were less likely to receive a MIS resection, to balance the two treatment groups, as would have happened by design in an RCT.
III) Finally, we estimated the effect of each surgical treatment on one-year mortality using mixed-effects logistic regression. We predicted two outcomes for each individual, one for each surgical treatment, defining the individual mortality risk difference caused by exposure to MIS resection. We calculated the ‘average treatment effect’ (ATE) as the average of each individual's risk difference in one-year mortality. An ATE of zero indicates no causal effect of surgical approach on one-year mortality; negative ATE shows a reduction in mortality due to MIS resection.

When using IPW-RA to account for the fact that patients can only receive one type of surgical treatment, the following assumptions are required to obtain an unbiased estimate of the causal effect of surgical approach on one-year mortality: no unmeasured confounders, correct model specification, positivity, conditional exchangeability and consistency. These are discussed in Supplementary Table S1.

Additional sub-analyses were stratified by stage at diagnosis. All analysis were performed in Stata 18.0.

### Ethics

The authors have obtained the ethical and statutory approvals required for this research (PIAG 1–05(c)/2007); ethical approval updated October 2021 (REC 21/LO/0552).

### Role of the funding source

The funding source had no role in collection, analysis, study design, interpretation of the data, writing of the report and decision to submit. The corresponding author had full access to all of the data and the final responsibility to submit for publication.

## Results

### Descriptive analysis and association of MIS vs OS resection with patient/tumour characteristics

Of 52,641 colon cancer patients diagnosed in England in 2021–2022, 21,931 (median age: 72.2 years, range: 19–96, of whom 10,362 women (47.2%)) with stage I-III disease were included in the analysis, all diagnosed through elective routes (Fig. 1, Table 1). The included cancers were located as follows: caecum: 4,729; ascending colon: 4,382; hepatic flexure: 1,373; transverse colon: 2,179; splenic flexure: 711; descending colon: 947; sigmoid colon: 7,457; unspecified colon: 153.

**Table 1:** Characteristics of 21,931 patients with Stage I-III colon cancer undergoing elective resection, England, 2021–2022.

	n (column %)		Open or MIS resection, n (row %)			
			Open		Minimally invasive	
All patients	21,931	100.0	5660	25.8	16,271	74.2
**Age group**^a^						
<55	1950	8.9	484	24.8	1466	75.2
55–64	4383	20.0	1076	24.5	3307	75.5
65–74	7517	34.3	1868	24.9	5649	75.1
75–84	6603	30.1	1810	27.4	4793	72.6
85+	1478	6.7	422	28.6	1056	71.4
**Sex**^a^						
Men	11,569	52.8	3200	27.7	8369	72.3
Women	10,362	47.2	2460	23.7	7902	76.3
**Deprivation quintile**^a^						
1 (least deprived)	5284	24.1	1219	23.1	4065	76.9
2	5304	24.2	1368	25.8	3936	74.2
3	4569	20.8	1180	25.8	3389	74.2
4	3766	17.2	1017	27.0	2749	73.0
5 (most deprived)	3008	13.7	876	29.1	2132	70.9
**Major comorbidity status**^a^						
None	15,049	68.6	3626	24.1	11,423	75.9
1 or more	6882	31.4	2034	29.6	4848	70.4
**Hospital frailty score (frailty risk category)**^a^						
0–4 (low)	19,609	89.4	4813	24.5	14,796	75.5
5–14 (moderate)	2028	9.2	752	37.1	1276	62.9
15+ (high)	294	1.3	95	32.3	199	67.7
**Cancer stage**^a^						
I	5393	24.6	1107	20.5	4286	79.5
II	8760	39.9	2302	26.3	6458	73.7
III	7778	35.5	2251	28.9	5527	71.1
**Vital status one year after surgical resection (column %)**^a^						
Alive	21,023	95.9	5224	92.3	15,799	97.1
Dead	908	4.1	436	7.7	472	2.9

a There was strong evidence of association between surgical approach and each of the patient/tumour characteristics (Pearson Chi-square p-value <0.001).

MIS resection was attempted in 18,264 (83.3% of 21,931) patients and completed in 16,271 (74.2%), including 1670 robotic resections. 5660 patients (25.8% of 21,931) underwent OS resection (including 1993 converted from MIS). Among the completed MIS resections, there were higher proportions in women (76.3%, 7902 of 10,362) than in men (72.3%, 8369 of 11,569) (Table 1), and similar proportions in 2021 and 2022 (73% and 75%). The proportion of patients with completed MIS resection ranged 68–83% (IQR) between individual NHS Trusts. The proportion of patients undergoing MIS resection decreased with increasing age (75.5%, 3307 of 4383 55–64 years to 71.4%, 1056 of 1478 85+ years at diagnosis), stage (79.5%, 4286 of 5393 stage I vs 71.1%, 5527 of 7778 stage III), deprivation (76.9%, 4065 of 5284 least vs 70.9%, 2132 of 3008 most deprived), comorbidity (75.9%, 11,423 of 15,049 patients with no comorbidities vs 70.4%, 4848 of 6882 patients with one or more major comorbidity) and frailty (75.5%, 14,796 of 19,609 low frail risk vs 67.7%, 199 of 294 high frail risk patients) (Table 1). Data on race or ethnicity were not available for this study.

**Fig. 2:**
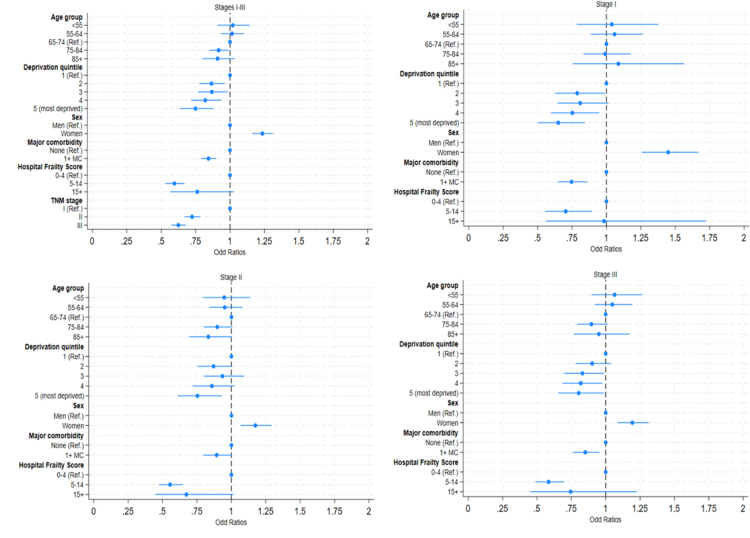
**Association between MIS resection vs OS resection and patient or tumour characteristics for stage I-III colon cancer patients undergoing elective resection, England, 2021–2022.** Notes: Results adjusted for age at diagnosis, deprivation quintile, sex, major comorbidity status, hospital frailty score and cancer stage, and for the clustering of standard errors by NHS Trusts. Full circles represent the Odds Ratios, the bars represent the 95% Confidence Intervals around each Odds Ratio.

After adjusting for age at diagnosis, sex, deprivation quintile, major comorbidity status and frailty risk category (Fig. 2), the Odd Ratios (ORs) for MIS resection were generally lower for moderate or high frailty risk than low frailty risk (Odds Ratio [OR]: moderate risk: 0.60, confidence intervals [95% CI] 0.53–0.67; high risk: 0.76, 95% CI 0.56–1.03), in more deprived than least deprived (OR: 0.75, 95% CI 0.64–0.88), in those with more advanced stage compared to stage I disease (OR stage III: 0.62, 95% CI 0.58–0.68; and OR stage II: 0.72, 95% CI 0.67–0.78), and in those with major comorbidities than those with no comorbidities (OR: 0.84, 95% CI 0.79–0.90). Women were 23% more likely to undergo MIS compared to men (OR: 1.23, 95% CI 1.16–1.31) (Fig. 2). Results were broadly similar in 21 NHS Trusts carrying out high volumes of MIS resections (>117, 4th quartile of MIS resections) and in 58 carrying out lower volumes (<57, 1st quartile of MIS resections), except concerning age: older patients in high-volume Trusts had equal or greater odds of MIS resection compared with younger patients (Supplementary Figure S1).

In the stage-stratified analyses, the effects of sex, deprivation, comorbidity and frailty risk category were similar to the overall analysis. For stage I patients, those with moderate frailty risk were 30% less likely to undergo MIS resection than those with low frailty risk (OR: 0.70, 95% CI 0.55–0.89). Those in the most deprived quintile were 35% less likely to undergo MIS resection than the least deprived (OR: 0.65, 95% CI 0.50–0.84) (Fig. 2). Those with major comorbidities were 25% less likely to undergo MIS resection that those with no major comorbidities (OR: 0.75, 95% CI 0.65–0.86). Women were 45% more likely to undergo MIS resection compared to men (OR: 1.45, 95% CI 1.26–1.67). Similar trends were seen in patients with stages II and III disease (Fig. 2).

Overall, 4.1% of patients died within one year after surgery (908 out of 21,931 patients, Table 1). One-year-mortality was higher in patients receiving OS (7.7%, 436 out of 5660 patients) than MIS resections (2.9%, 472 out of 16,271 patients). Mortality within 90 days after OS resection was 3.1% (175 patients) vs 1.1% (171 patients) after MIS resection. Of the deaths after OS resection, 40% occurred within 90 days (175 out of 436 deaths); after MIS resection the figure was 36.0% (171 out of 472 deaths; Supplementary Figure S2). The probability of surviving 2 years from the date of surgical resection was higher among patients undergoing MIS resection (93.7%, 95% CI: 93.2; 94.1) than OS resection (86.3%, 95% CI: 85.2; 87.2; Supplementary Figure S2). For completeness, calculating on intention to treat basis (conversions included with MIS), one-year mortality for OS vs MIS resection was 8.5 vs 3.3%.

Four-fifths of patients presented at least one high-risk characteristic (78.5%) and their mortality was 8.6% with OS and 3.4% with MIS resections; the remaining one-fifth (21.5%) of patients had none of the high-risk features and their one-year mortality was 3.3% with OS and 1.2% with MIS resections (data not shown).

Among the 124 NHS Trusts, 70, 77, 93 and 98 had lower implementation of MIS resection (inequality) in patients who were over 65 years, or more deprived, or comorbid, or frail, respectively. In 20–43% of Trusts (varying by high-risk group), the proportion of MIS resections was higher in at least one of the disadvantaged groups. In 25 Trusts (20% of 124 Trusts), the proportion of MIS resections was higher among patients with moderate or high frailty scores than in less frail patients. In 31 Trusts (25% of 124 Trusts) there was no inequality regarding comorbidity (data not shown).

### Average treatment effect of MIS vs OS resection on one-year mortality

Considering the study population as a whole, the average treatment effect of MIS resection rather than OS resection was an absolute reduction in one-year mortality from 6.8% (OS resection, 95% CI 6.2–7.5) to 3.0% (MIS resection, 95% CI 2.8–3.3) (average absolute reduction −3.8% 95% CI −4.5, −3.1; Table 2).

**Table 2:** Average treatment effect on one-year mortality in an emulated trial of MIS vs OS resection for stage I-III colon cancer, England, 2021–2022.

	Age groups	Average mortality with OS	Lower 95% CI	Upper 95% CI	Average mortality with MIS	Lower 95% CI	Upper 95% CI	Average absolute reduction in mortality with MIS	Lower 95% CI	Upper 95% CI	Average relative reduction in mortality with MIS (%)
**All patients**	All	6.8	6.2	7.5	3.0	2.8	3.3	−3.8	−4.5	−3.1	55.9
**Age at diagnosis**	<55	2.9	1.4	4.3	0.3	0.0	0.6	−2.6	−4.1	−1.1	89.7
	55–64	3.4	2.5	4.4	1.2	0.8	1.6	−2.3	−3.3	−1.2	64.7
	65–74	5.1	4.3	6.0	2.6	2.2	3.0	−2.5	−3.5	−1.6	49.0
	75–84	10.2	9.0	11.4	5.0	4.4	5.5	−5.2	−6.6	−3.9	51.0
	85+	16.1	12.8	19.4	6.3	4.9	7.7	−9.8	−13.4	−6.2	60.9
**All patients 65+**											
Low frailty risk	65–74	4.5	3.6	5.4	2.3	1.9	2.7	−2.2	−3.2	−1.2	48.9
	75–84	9.4	8.1	10.8	3.9	3.3	4.6	−5.5	−6.9	−4.1	58.5
	85+	13.2	10.1	16.3	5.8	4.2	7.4	−7.4	−10.9	−3.9	56.1
Moderate and high frailty risk	65–74	12.5	8.9	16.0	5.9	3.5	8.3	−6.6	−10.3	−2.8	52.8
	75–84	14.4	10.7	18.1	10.8	8.4	13.2	−3.6	−8.0	0.7	25.0
	85+	26.2	17.7	34.8	8.0	4.3	11.8	−18.1	−27.3	−9.1	69.5
No major comorbidity	65–74	4.8	3.6	6.0	2.1	1.6	2.6	−2.7	−4.0	−1.3	56.3
	75–84	9.2	7.5	10.8	3.7	3.0	4.4	−5.5	−7.2	−3.8	59.8
	85+	13.8	9.8	17.7	6.3	4.4	8.2	−7.4	−12.0	−2.9	54.3
Major comorbidity	65–74	5.9	4.3	7.6	3.8	2.8	4.7	−2.2	−3.8	−0.5	35.6
	75–84	11.7	9.4	14.1	6.9	5.8	8.0	−4.8	−7.3	−2.3	41.0
	85+	19.4	14.2	24.6	6.4	4.3	8.4	−13.0	−18.5	−7.6	67.0
**Patients 65+ with cancer stage III**											
Low frailty risk	65–74	6.5	4.6	8.3	4.0	3.0	5.0	−2.5	−4.6	−0.4	38.5
	75–84	15.5	12.6	18.4	4.8	3.7	5.9	−10.7	−13.7	−7.7	69.0
	85+	20.9	13.5	28.2	9.8	5.9	13.7	−11.1	−19.5	−2.6	53.1
Moderate and high frailty risk	65–74	21.4	12.9	29.8	10.9	5.2	16.7	−10.5	−20.4	−0.5	49.1
	75–84	25.3	17.0	33.6	16.3	11.5	21.1	−9.0	−19.2	1.1	35.6
	85+	26.6	14.2	39.1	10.2	3.6	16.8	−16.5	−30.5	−2.4	61.7
No major comorbidity	65–74	7.5	5.1	9.8	3.8	2.8	4.9	−3.6	−6.3	−0.9	49.3
	75–84	14.8	11.4	18.3	5.4	4.2	6.7	−9.4	−12.8	−6.0	63.5
	85+	18.8	10.6	27.1	10.4	5.8	15.0	−8.5	−18.2	1.2	44.7
Major comorbidity	65–74	8.0	5.1	10.8	6.2	4.0	8.3	−1.8	−5.3	1.6	22.5
	75–84	20.1	15.8	24.3	8.0	5.7	10.3	−12.1	−16.6	−7.5	60.2
	85+	26.9	16.4	37.4	9.5	4.8	14.3	−17.4	−28.8	−5.8	64.7
**Patients 65+ in deprivation quintiles 3–5 (most deprived)**											
Low frailty risk	65–74	4.5	3.1	6.0	2.6	2.0	3.2	−2.0	−3.6	−0.3	42.2
	75–84	10.1	8.0	12.2	4.5	3.6	5.3	−5.6	−8.0	−3.3	55.4
	85+	13.7	7.8	19.5	5.5	3.1	7.9	−8.1	−14.5	−1.8	59.9
Moderate and high frailty risk	65–74	13.2	7.9	18.5	6.6	3.2	10.0	−6.7	−12.3	−1.0	50.0
	75–84	16.3	11.5	21.1	12.8	9.3	16.3	−3.5	−9.5	2.4	21.5
	85+	21.7	10.6	32.8	6.6	2.0	11.1	−15.1	−27.5	−2.8	69.6
No major comorbidity	65–74	5.0	3.2	6.7	2.2	1.6	2.9	−2.8	−4.7	−0.8	56.0
	75–84	10.4	7.8	12.9	4.1	3.0	5.1	−6.3	−9.0	−3.5	60.6
	85+	11.0	4.3	17.7	5.4	2.3	8.5	−5.6	−12.9	1.7	50.9
Major comorbidity	65–74	5.9	3.7	8.1	4.3	2.9	5.7	−1.6	−4.1	0.8	27.1
	75–84	12.1	9.5	14.8	8.2	6.6	9.8	−3.9	−7.2	−0.7	32.2
	85+	19.9	12.8	27.0	6.3	3.1	9.4	−13.6	−21.7	−5.6	68.3

Notes: MIS: Minimally invasive surgery; OS: open surgery. 95% CI: 95% confidence intervals.

**Fig. 3:**
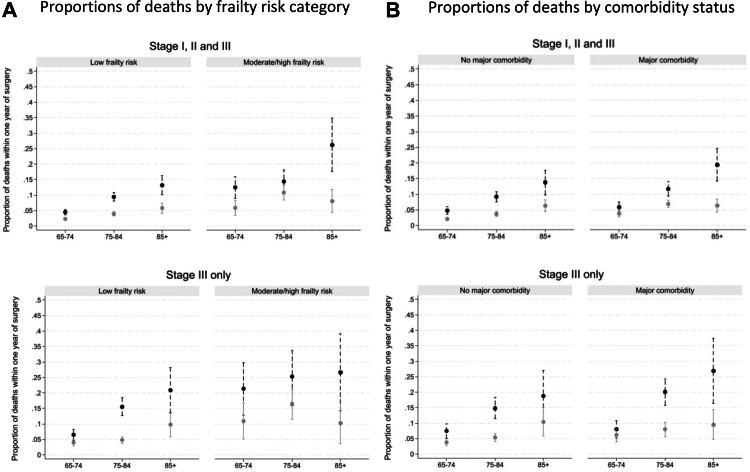
**Comparison of proportion of deaths in first year after surgery between MIS resection and OS resection (potential outcomes), in the overall study population (top panel) and in the subgroup of patients with cancer stage III (bottom panel), by frailty risk category (A) or comorbidity status (B)**. Notes: MIS: Minimally invasive surgery resection (grey); OS: open surgery resection (black). Full circles represent the proportions of deaths, the dashed bars represent the 95% Confidence Intervals around each proportion.

Examining potential interactions between high-risk factors, and considering all patients aged 65 and over, the beneficial effect of undergoing MIS rather than OS resection increased with increasing frailty score, regardless of deprivation (Table 2). Patients aged 85 years and older with moderate/high risk of frailty had the highest potential mortality following OS resection (26.2%, 95% CI 17.7–34.8) as well as the greatest absolute and relative reduction (improvement) in mortality when using MIS resection (−18.1%, 95% CI: −27.3, −9.1; Table 2, Fig. 3A). For patients aged 85 years and older, with stage III disease (Table 2, Fig. 3A) undergoing MIS resection led to an absolute 11.1% (95% CI −19.5, −2.6) and 16.5% (95% CI −30.5, −2.4) decrease in one-year mortality for low-frailty and moderate/high frailty risk, respectively. For patients aged 85 years and older, with stage III disease (Table 2, Fig. 3B) with major comorbidities, MIS resection led to an absolute 17.4% decrease in one-year mortality (95% CI −28.8, −5.8) compared with OS resection (9.5% for MIS vs 26.9% for OS).

## Discussion

In this observational study based on the English national cancer dataset, we used emulated trial methodology and the potential outcomes framework to investigate one-year mortality following elective MIS resection compared with OS resection for colon cancer. Elective MIS resection for Stage l-lll colon cancer led to a reduction in one-year mortality compared with OS resection. This benefit was greatest among patients conventionally considered “high risk” for surgical resection (patients who were old, frail, comorbid or from the most socioeconomically deprived areas). However, in 2021–2022, these patient groups were less likely to receive an MIS resection.

We confirm previous reports that patients who are old^15^^,^^33^^,^^34^ or comorbid^16^ benefit from MIS resection rather than OS resection for colon cancer, and show that patients from additional groups previously considered “high risk” for major surgery i.e. patients with a background of social deprivation or frailty, should also benefit from MIS rather than OS resection. We chose one-year mortality as our endpoint because deaths related to colon cancer resection may occur beyond the date of discharge or the conventional 90-day post-surgery endpoint. Mortality, one year after MIS resection, was less than half that observed after OS resection, whether analysed on an intention to treat basis or in terms of MIS resections completed.

Although RCTs are the gold standard in establishing the benefits of one treatment over another, they may not adequately represent the spectrum of patients who are older, frail or comorbid. National observational datasets allow insights into the effectiveness of treatments on outcomes across whole populations including patients who might be unsuitable for inclusion in trials.^35^ Emulated trial methodology provides methods for formally investigating causal effects using observational data, by reducing bias from non-randomised comparisons.^26^^,^^36^ This novel approach mimics the ideal RCT to answer the research question of interest, stipulating eligibility, inclusion and exclusion criteria using the selected observational dataset.^37^ Provided key confounders are measured and assumptions are justified, emulated trials based on observational data can provide rigorous evidence of treatment benefit and can support causal inferences about treatment effects.^38^^,^^39^ The present emulated trial focused on whether elective MIS resection improves mortality at 1 year compared with OS resection. Operations were categorised as MIS or OS resections based on treatment delivered rather than intention-to-treat, reflecting the type of resection actually experienced by patients. We limited the trial to patients undergoing elective resections for colon cancer. Elective patients are discussed in an MDT meeting, and their surgery is planned and delivered by specialist MDT colorectal surgeons. Including emergency patients would have introduced additional confounding factors comprising varying levels of acute illness, a wider spectrum of skill among the operating surgeons and potentially variable availability of operating equipment or theatre teams to support emergency MIS resection. The trial might have benefited had data on BMI, previous abdominal surgery and reasons for deciding OS resection been available. However, the strength of an emulated trial is its ability to identify the effectiveness of an intervention, such as MIS resection, capturing variation in implementation as happens in the “real world”, obtained from observational data.

In the emulated trial, completed MIS resection reduced mortality one year post elective surgery for colon cancer to less than half that seen after OS resection. This implies that the better results seen after MIS resection cannot be (solely) attributed to the way in which patients were selected for MIS vs OS resection. The findings support previous observational reports.^9^, ^10^, ^11^ While we cannot say that reduced one-year mortality is entirely attributable to resection technique, the results suggest that completed MIS resection contributes to the reduction and that higher-risk patients who are selected for surgery benefit from MIS resection.

Whether MIS resection is, in fact, feasible in more patients from “high risk” groups than currently receive it, is a central consideration. Small numbers of higher-risk patients may not be able to tolerate pneumoperitoneum or longer operating times and may be best served by OS resection or conversion to OS resection. Others, as for lower-risk patients, may require OS resection because of very extensive adhesions from previous surgery, because they need multivisceral resection, or following conversion from initial MIS approach. However, given that by 2018, in the Netherlands, as many as 85–90% of elective T1-3 colon cancer resections were completed using an MIS approach, and noting the high prevalence of at least one high-risk factor in our patient population (78.5%), it is likely that many more high-risk as well as lower-risk patients are indeed suitable for MIS resection.^40^^,^^41^ We report that up to two fifths of Trusts carried out greater proportions of MIS resection in particular ‘high-risk’ patient groups. We see this disparity as evidence that inequalities in implementation of MIS resection in ‘high-risk’ patient groups are largely related to issues at Trust level. Variation in rates of MIS colon resection in the USA, have been reported to be predominantly attributable to surgeon variation, rather than patient variation.^42^

In England, surgery for colorectal cancer is undergoing rapid transformational change, as open resection is increasingly replaced by laparoscopic and more recently, robotic MIS resection. We confirm previous reports that, as recently as 2021–2022, patients having MIS resection in England were likely to be younger, from areas of less social deprivation, with less advanced disease, less comorbidity and less frailty than those undergoing OS resection.^10^^,^^11^^,^^22^^,^^23^ Our results show that this inequality in delivery of MIS resection negatively impacts one-year survival among higher-risk patient groups. Previous authors,^22^^,^^23^ and sequential NBOCA reports,^20^^,^^43^^,^^44^ indicate persisting major regional variation in the proportion of patients with bowel cancer (colon and rectum) having MIS vs OS resection in England.^43^ For 2021–2022, although NBOCA documented a laparoscopic approach in 74% of colorectal cancer resections, the range was 60–84% between the 11 (regional) Cancer Alliances.^20^ Such major geographic variation in use of MIS resection indicates ongoing regional inequalities in treatment of colon cancer in England, which, in the context of the present results, are likely to be most detrimental to patients who are old, frail, comorbid or deprived.

These observations have policy implications. Given that NICE gave qualified approval to MIS resection as long ago as 2006, continuing regional inequalities in treatment of colon cancer by MIS resection in England suggest examination of individual colorectal MDTs to determine the causes and to generate potential solutions.^18^ NBOCA has responded by tasking NHS Trusts to complete >50% of resections for colorectal cancer via a laparoscopic or robotic approach.^44^ Our results, in Trusts doing the most resections, suggest that increasing implementation of MIS resection will need to be associated with changes in decision-making to ensure that higher-risk patients also benefit.

Further, if MIS resection reduces postoperative mortality in high-risk groups, then efforts to increase MIS resection in high-risk groups may render some patients eligible for surgery who would otherwise be considered too unfit. Recognising that surgical resection is the most effective treatment for localised colon cancer and that resection rates are lower in England than in many nations with similar healthcare systems, increasing the proportion of deprived, frail, comorbid or older patients safely undergoing resection has the potential to reduce inequalities in the care of colon cancer and improve survival for colon cancer patients in England.

Additional outcomes should be considered in determining whether increased use of MIS resection in high-risk patient groups should become policy. These include likelihood of return to previous levels of independence, Patient-Reported Outcomes (PROMS) and patient's quality of life through their cancer journey as well as cost and resource implications (including availability of sufficient skilled surgeons).

We report potential limitations: We included only patients accepted for and undergoing elective resection and have not considered patients undergoing emergency operation. It is possible that proportionately fewer patients who were deprived, old, frail, and comorbid were accepted for surgery, but this is beyond the scope of this study. Additionally, some MDTs may have had policies for managing particular patient groups by OS resection. Partially observed or missing baseline variables, under-ascertainment of clinical factors, from secondary care records (unmeasured confounding) could impact our results: For example, comorbidity and frailty may not be documented in people who have not previously required hospital admission (HES) or where entries in the clinical record have been inadequate. This is a well-acknowledged challenge of target trial emulation.^45^^,^^46^

In conclusion, in patients with colon cancer, an emulated trial shows that MIS resection reduces one-year-mortality compared with OS resection. Patients who are old, frail or comorbid have greatest reductions in one-year mortality after MIS resection but remain at most risk of not receiving it. The results lend support to MIS resection as the standard of care for colon cancer resection across England, including for “high risk” patients. The present study shows how introduction of innovative treatments may generate new inequalities in cancer care. Regarding MIS resection for colon cancer, the NHS needs to act to ensure equity of treatment for all patients. Such an approach has the potential to improve survival from colon cancer in England.

## Contributors

Conceptualisation (SBM, BR), access and verification of the (raw) data (SBM, CM, MQ), data curation (CM, SBM, MQ), formal analysis (SBM, CM, MQ), funding acquisition (BR), investigation (DOL, SBM, BR), methodology (CM, CL, MQ), project administration, resources, software, supervision (BR, MQ), validation (CM, MQ), visualisation (CM, MQ), writing—original draft (SBM, DOL, CM, MQ), and writing–review & editing (DOL, CM, SBM, CL, BR, MQ). All authors read and approved the final version of the manuscript. CM and MQ have verified the underlying data.

## Data sharing statement

The data used for this study are the English National Cancer Registry data and the Hospital Episode Statistics (HES). This data consists of patient information and as such, it is protected under the Data Protection Act 1998 and GDPR 2018 and cannot be made available as open data. Formal requests for release of the data can be made to the data custodian, NHS England. The researchers have obtained ethical and statutory approvals required for accessing sensitive data.

## Declaration of interests

The authors declare no competing interests.
