# Supplementary material for: Minimally invasive surgical resection reduces one-year mortality, especially in high-risk colon cancer patients: an emulated trial
Source: eClinicalMedicine. 2026 Feb 2;92:103683. doi: 10.1016/j.eclinm.2025.103683 (PMC12947640; doi:10.1016/j.eclinm.2025.103683)
Supplement: Supplementary Figures and Tables [file mmc1.docx]

Supplementary Table 1: Specification and emulation of a target trial of surgical approach (MIS *vs*. OS) in colon cancer patients diagnosed in 2021-2022 in England.

| **Component** |  | **Randomised Controlled Trial (Target trial)** |  | **Emulated trial using real-world data** |
| --- | --- | --- | --- | --- |
| **Design** |  | Multicentre open-label two-parallel arm superiority randomised trial. |  | Hypothetical randomised controlled trial using retrospectively -collected national population data |
| **Aim** |  | Estimate the effect of completed MIS versus OS resection performed within six months of colon carcinoma diagnosis, on 1-year mortality |  | Estimate the effect of completed MIS versus OS resection performed within six months of colon carcinoma diagnosis, on 1-year mortality |
| **Eligibility** |  | Colon cancer patients diagnosed at age 15-99 years, at TNM stages I-III, who receive elective colon cancer resection during 2021 and 2022 |  | Colon cancer patients diagnosed at age 15-99 years, at TNM stages I-III, who have a record of elective colon cancer resection during 2021 and 2022 in the six months after diagnosis |
| **Exclusions** |  | Patients with endoscopic procedure only, patients who previously underwent major bowel surgery;  Patients treated in independent hospitals, in Trusts with no records of either MIS or OS in 2021-22 or in Trust with 10 or fewer resections in 2021-2022 |  | Patients with endoscopic procedure only, patients who previously underwent major bowel surgery;  Patients treated in independent hospitals, in Trusts with no records of either MIS or OS in 2021-22 or in Trust with 10 or fewer resections in 2021-2022 |
| **Treatment strategies** |  | 1. MIS approach and resection  2. OS resection |  | 1. All patients whose surgery was completed as MIS resection  2. All patients whose surgery was completed as OS resection |
| **Treatment assignment** |  | Patients are randomly assigned to either strategy. |  | Patients are non-randomly assigned to a treatment strategy based on data on “treatment delivered” rather than “intention to treat”. Randomisation is “emulated” using inverse probability of treatment weights. |
| **Treatment implementation** |  | MIS/OS resection according to randomisation protocol. |  | MIS/OS resection, according to emulated randomisation protocol. |
| **Outcome** |  | Death from all causes within a year of diagnosis. |  | Death from all causes within a year of diagnosis |
| **Type of outcome** |  | Binary |  | Binary |
| **Follow up** |  | Alive or dead 12 months post resection |  | Alive or dead 12 months post resection |
| **Censoring** |  | None |  | None |
| **Adjustment variables** |  | Age at diagnosis (15-99 years), sex (1,2), deprivation quintiles (1-5), stage at diagnosis (1,2,3), Major comorbidity (0,1), frailty. |  | Age at diagnosis (15-99 years), sex (1,2), deprivation quintiles (1-5), stage at diagnosis (1,2,3), Major comorbidity (0,1), frailty. |
| **Causal contrast** |  | *Per protocol* |  | *Per protocol* |
| **Estimand** |  | Risk difference in one-year mortality between arms |  | Risk difference in one-year mortality between arms |

Causal inference assumptions:

There are four key assumptions needed to identify causal treatment effects from observational data.^19^

Non-interference: The allocation of one patient to one treatment does not affect allocation of another patient; not problematic in our setting

Consistency: A patient’s *potential* outcome for a given treatment (MIS or OS) is the *actual* outcome observed for that patient, had they received that treatment. This is the same as stating that there are not multiple versions of a specific treatment. In the emulated trial the purpose was to compare 1 year mortality after open surgical resection versus MIS resection (however achieved). The precise definition of our treatment (minimally invasive surgical resection = single category incorporating laparoscopic, robotic, single port etc) improves the plausibility of that assumption.

Positivity: Each patient could receive either treatment. To reduce the risk of issues with this assumption, we restricted the sample to patients likely to be eligible for both approaches (i.e., those with non-metastatic disease and diagnosed through a non-emergency route) and to Trusts that carry out both surgical approaches.

Conditional exchangeability: In an RCT, this assumption is usually met by randomising the treatment allocation (using a procedure that is not biased based on covariate information), so that patients may be “swapped” between treatment groups without changing the estimated treatment effect. In an emulated trial using observational data, we try to meet that assumption by adjusting/conditioning on potential confounding introduced by some variables, so that if patients were to be “swapped” between treatment groups, the estimated treatment effect would not be affected, i.e. the outcome is independent. To help with this assumption, covariates to adjust for were carefully selected using a Directed Acyclic Graph (DAG).

When drawing causal effects from observational data, the validity of these assumptions needs to be considered. Although in the present context, regional variations in the distribution of MIS, owing to surgeons’ training and expertise and institutional experience and volume, might compromise some of them, we believe the impact of these violations on our results is minimal. The implementation of MIS resection might vary between Trusts, impacting the consistency assumption, however it is still key (and valid) to consider the overall effect of MIS resection from a policy viewpoint, capturing natural variability, hence estimating a more pragmatic effect. We ensured positivity by selecting Trusts offering both types of surgery. We ensured conditional exchangeability by adjusting for individual confounding variables and between-Trust heterogeneity via random effects in the statistical models.

Supplementary Table 2: STROBE Statement—checklist of items that should be included in reports of observational studies

|  | Item No | Recommendation |  |
| --- | --- | --- | --- |
| **Title and abstract** | 1 | (*a*) Indicate the study’s design with a commonly used term in the title or the abstract | Title |
|  |  | (*b*) Provide in the abstract an informative and balanced summary of what was done and what was found | Summary: Methods and findings |
| Introduction | | |  |
| Background/rationale | 2 | Explain the scientific background and rationale for the investigation being reported | Introduction |
| Objectives | 3 | State specific objectives, including any prespecified hypotheses | Last paragraph of introduction  Aim in methods section |
| Methods | | |  |
| Study design | 4 | Present key elements of study design early in the paper | Design of the emulated trial in the methods section |
| Setting | 5 | Describe the setting, locations, and relevant dates, including periods of recruitment, exposure, follow-up, and data collection | Data sources in the methods section |
| Participants | 6 | (*a*) *Cohort study*—Give the eligibility criteria, and the sources and methods of selection of participants. Describe methods of follow-up  *Case-control study*—Give the eligibility criteria, and the sources and methods of case ascertainment and control selection. Give the rationale for the choice of cases and controls  *Cross-sectional study*—Give the eligibility criteria, and the sources and methods of selection of participants | Data sources in the methods section  Eligibility and exclusion criteria in the methods section |
|  |  | (*b*) *Cohort study*—For matched studies, give matching criteria and number of exposed and unexposed  *Case-control study*—For matched studies, give matching criteria and the number of controls per case |  |
| Variables | 7 | Clearly define all outcomes, exposures, predictors, potential confounders, and effect modifiers. Give diagnostic criteria, if applicable | Treatments and assignment in methods section  Adjustment variables in methods section |
| Data sources/ measurement | 8* | For each variable of interest, give sources of data and details of methods of assessment (measurement). Describe comparability of assessment methods if there is more than one group | Treatments and assignment in methods section  Adjustment variables in methods section |
| Bias | 9 | Describe any efforts to address potential sources of bias | Analyses in methods section |
| Study size | 10 | Explain how the study size was arrived at | First paragraph of results section |
| Quantitative variables | 11 | Explain how quantitative variables were handled in the analyses. If applicable, describe which groupings were chosen and why | Adjustment variables in methods section |
| Statistical methods | 12 | (*a*) Describe all statistical methods, including those used to control for confounding | Analyses in methods section |
|  |  | (*b*) Describe any methods used to examine subgroups and interactions | Analyses in methods section |
|  |  | (*c*) Explain how missing data were addressed | Analyses in methods section |
|  |  | (*d*) *Cohort study*—If applicable, explain how loss to follow-up was addressed  *Case-control study*—If applicable, explain how matching of cases and controls was addressed  *Cross-sectional study*—If applicable, describe analytical methods taking account of sampling strategy | NA |
|  |  | (*e*) Describe any sensitivity analyses | Analyses in methods section |

Continued on next page

| Results | | |  |
| --- | --- | --- | --- |
| Participants | 13* | (a) Report numbers of individuals at each stage of study—eg numbers potentially eligible, examined for eligibility, confirmed eligible, included in the study, completing follow-up, and analysed | First paragraph of results section |
|  |  | (b) Give reasons for non-participation at each stage | NA |
|  |  | (c) Consider use of a flow diagram | Figure 1 |
| Descriptive data | 14* | (a) Give characteristics of study participants (eg demographic, clinical, social) and information on exposures and potential confounders | Table 1 |
|  |  | (b) Indicate number of participants with missing data for each variable of interest | Figure 1  First paragraph of results section |
|  |  | (c) *Cohort study*—Summarise follow-up time (eg, average and total amount) | Not provided as such  Supplementary Figure 2 provides Kaplan Meier curves |
| Outcome data | 15* | *Cohort study*—Report numbers of outcome events or summary measures over time | Table 1 |
|  |  | *Case-control study—*Report numbers in each exposure category, or summary measures of exposure | *NA* |
|  |  | *Cross-sectional study—*Report numbers of outcome events or summary measures | *NA* |
| Main results | 16 | (*a*) Give unadjusted estimates and, if applicable, confounder-adjusted estimates and their precision (eg, 95% confidence interval). Make clear which confounders were adjusted for and why they were included | Table 2 |
|  |  | (*b*) Report category boundaries when continuous variables were categorized | All tables and throughout results section |
|  |  | (*c*) If relevant, consider translating estimates of relative risk into absolute risk for a meaningful time period | Table 2 |
| Other analyses | 17 | Report other analyses done—eg analyses of subgroups and interactions, and sensitivity analyses | Table 2 and Figure 3 |
| Discussion | | |  |
| Key results | 18 | Summarise key results with reference to study objectives | Start of Discussion section |
| Limitations | 19 | Discuss limitations of the study, taking into account sources of potential bias or imprecision. Discuss both direction and magnitude of any potential bias | Last paragraph of the discussion section |
| Interpretation | 20 | Give a cautious overall interpretation of results considering objectives, limitations, multiplicity of analyses, results from similar studies, and other relevant evidence | Throughout the discussion section |
| Generalisability | 21 | Discuss the generalisability (external validity) of the study results | Throughout the discussion section |
| Other information | | |  |
| Funding | 22 | Give the source of funding and the role of the funders for the present study and, if applicable, for the original study on which the present article is based | Available |

*Give information separately for cases and controls in case-control studies and, if applicable, for exposed and unexposed groups in cohort and cross-sectional studies.

**Supplementary Figure 1. Association between MIS vs. OS resection and patient or tumour characteristics for Stage I-III colon cancer, by number of MIS performed in a NHS Trust, England, 2021-2022**

1. **NHS Trusts in first quartile (less than 57 MIS per year) B. NHS Trusts in fourth quartile (117 or more MIS per year)**

**
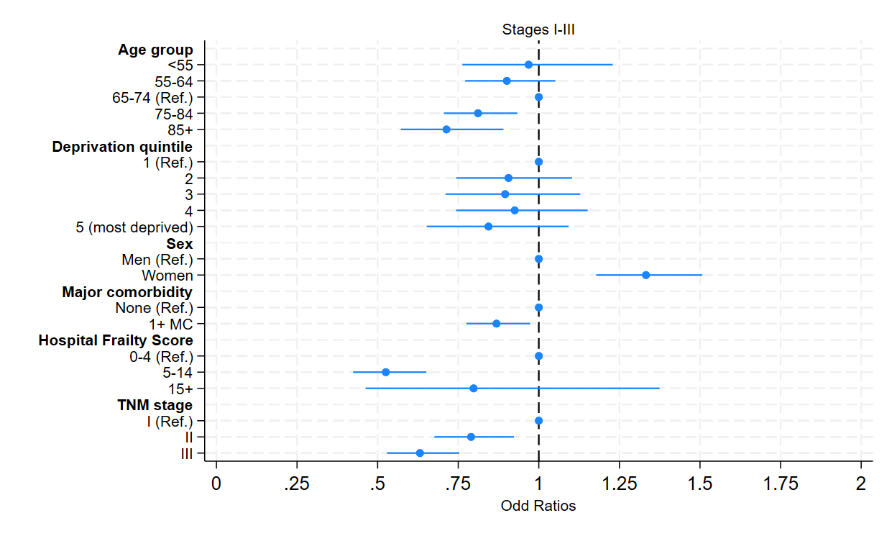

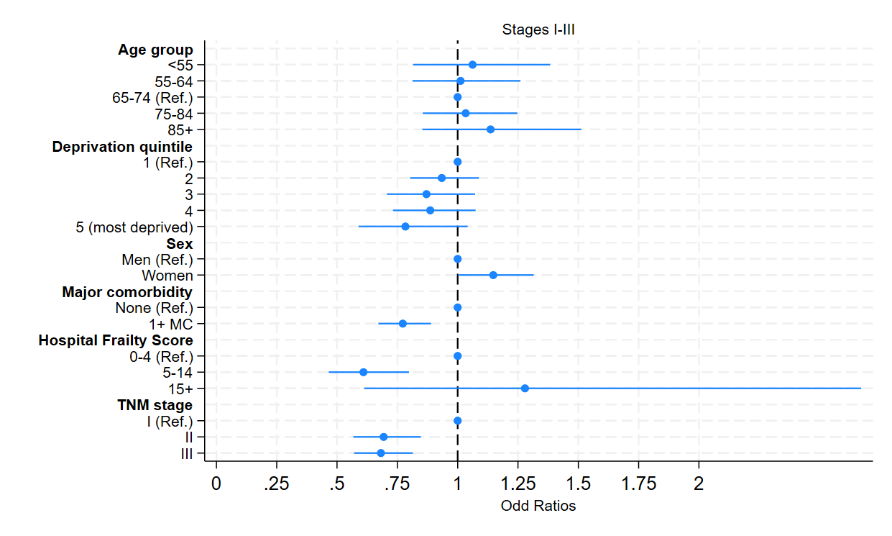
**

**Supplementary Figure 2. Kaplan Meier survival estimates by resection type, patients with colon cancer diagnosed in 2021-2022 in England**

**
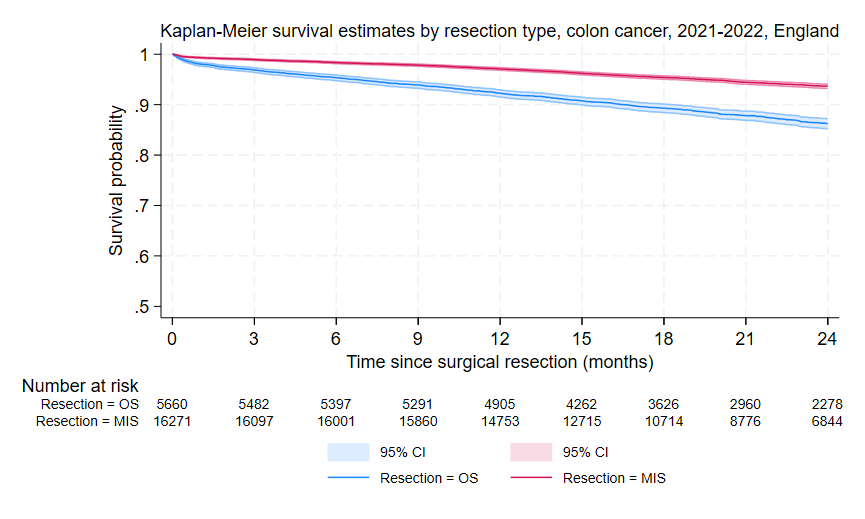
**
